# Quality of work life for health professions in Colombia’s adult critical care: An integrative analysis

**DOI:** 10.1186/s12913-024-10780-z

**Published:** 2024-05-03

**Authors:** Laura del Pilar Quiñones-Rozo, Gladys Eugenia Canaval-Erazo

**Affiliations:** PROMESA research group, Escuela de Enfermer?a, Facultad de Salud, Universidad del Valle, Cali, Colombia

**Keywords:** Health workforce, Critical care, Workplace violence, Spirituality, Occupational risks, Compassion fatigue, Quality of life, Job

## Abstract

**Background:**

Health professionals in Colombian and many parts of the world, in some cases, work in precarious conditions and intend to migrate to other countries in search of better living conditions for themselves and their families, which results in inadequate distribution worldwide and in the quality of care throughout the health system, which can ultimately influence the quality of life of patients in their health-disease processes.

**Purpose:**

Describe in depth what quality of life at work is like for the health workforce in adult critical care (ACC).

**Methods:**

This is an investigation of convergent parallel mixed methods approach that are integrated by means of a matrix in terms of convergence, divergence, and complementarity. Two methods are used: a transversal analytical method in which three instruments were applied to 209 participants to study the relationship between Quality of Life at Work, exposure to psychosocial risks, compassion fatigue and the intention to rotate; other than from the experiences narrated by 10 Human Talent in Health explore organizational practices in critical care.

**Results:**

The dimension of quality of work life with the greatest dissatisfied was the management of free time (77%), the most compromised psychosocial risk was the pace of work (84%). They have high compassion satisfaction (67%) and there is an intention to migrate to another country (66%). The narrative results in discrimination/harassment as normalized practices and faceless spirituality. The integration of mixed methods shows convergence between the use of the instrument that measures quality of life at work and the narratives of the participants, complementarity with the other instruments, and divergence regarding the intention to rotate to another health institution.

**Conclusion:**

The positive trend that converges with the two approaches is that of safety at work and well-being achieved through work, embodied in the constant updating of technology and care protocols, experience time, balance between salary and work effort, staffing and supplies, and disconnection with work.

**Supplementary Information:**

The online version contains supplementary material available at 10.1186/s12913-024-10780-z.

## Introduction

In the Adult Critical Care - ACC, the work environment is highly demanding and complex due to the seriousness of the patients, the need to use sophisticated equipment, the constant clinical assessments and the complex procedures that are performed. At the same time, the health workforce - HWF in the ACCs is under the direct influence of factors that affect their Quality of Life at Work - QWL [1–4]. They are also exposed to multiple situations that have repercussions at unsuspected levels in their physical, emotional, spiritual, social, and economic dimensions, among others. The QWL and the HWF, both in Colombia and in other countries around the world, are directly or indirectly affected by elements of a political and administrative nature, especially for those who are dedicated to providing care in the ACCs [5, 6].

In Colombia, in the last 20 years, there have been more than 10 modifications to the norms, decrees and resolutions regarding the minimum essential requirements for the authorization and operation of Health Service Provider Institutions (HSPI) that regulate the minimum number of health care personnel per bed in the ACC [7]. However, these modifications have gaps, and there is no strict control of their compliance, which allows the HSPI to make decisions that generate poorly distributed workloads that put the health system in general at risk and consequently affect the quality of life at work of human talent due to the apparent ineffectiveness of the system and the invisibility of the needs of the HWF.

The effects on the QWL of professionals who dedicate their lives to saving those of others have an impact on the quality of care for system users, which goes against Goal 8 of Sustainable Development, which proposes dignified and decent work as a goal for all.

Some studies on the QWL of the HWF propose mixed methodological designs to investigate this phenomenon, with special interest in nursing staff [8, 9], and frequently show results separately without integrating their data [10, 11]. Since QWL is multidimensional its measurement requires a different perspective to be studied.

The objective of this research was to describe in depth the Quality of Life at Work in CCA in a region of a Colombian geographic sector, to establish reflective approaches on the motivations that drive the HWF to provide its services despite the presence of negative factors that They can affect them directly or indirectly and on which they can intervene in the future.

## Methods

This is a convergent parallel mixed methods approach investigation that integrates the narrative [12] with the descriptive results of a cross-sectional research approach using a matrix in terms of convergence, divergence and complementarity. The recommendations given in the Mixed Methods Appraisal Tool (MMAT) checklist, version 2018 [13] are followed.

### Study design and sample

Mixed study of concurrent triangulation that combines the results of the analytical cross-sectional quantitative approach with the explanatory power of the qualitative approach using narrative, with cross-validation between the two approaches.

The sample used in the quantitative approach was 209 health professionals working in adult critical care units, with a minimum of one year of experience, extracted by two-stage stratification and simple random sampling (94% confidence; 6% error). In parallel, 10 participants were invited through a network approach and snowball technique as key informants. All participants were asked for informed consent.

### Instruments used in the quantitative approach

The questionnaire that measures Quality of Life at Work - QWL GOHISALO short version original language in Spanish that evaluates seven dimensions [14], the questionnaire that measures fatigue and compassion satisfaction with the Quality of Life Scale was used. Professional acronym in English “Professional Quality of Life Scale” ProQoL version IV [15, 16] and the Copenhagen Psychosocial Questionnaire - short version CopSoQ to measure its 15 dimensions [17–21]. The three highly reliable instruments with reported Cronbach’s alpha values of 0.95, 0.8 and 0.9 respectively, use a Likert-type scale and have been translated into several languages and validated in multiple languages.

In addition to the previous battery of questions five questions are added about the frequency with which you think about changing work environments, based on previous research on turnover intention [22, 23]. They measure the intention to change to another service, change to another institution, migrate to another city, migrate to another country, or change profession to independent work, through a scale of 1 to 5 where 1 = never, 2 = sometimes./year, 3 = with some frequency/month, 4 = almost always/week and 5 = always/every day, in addition to the population characterization questions.

### Techniques in the qualitative approach

Guiding questions are written to the semi-structured interviews adapted from other authors [24] and are found in the complementary material. Interviews were used via a virtual platform, recorded and carried out outside working hours in their homes, and transcribed using codes to ensure confidentiality. Each participant was also given an electronic notebook, in Word format, shared on a drive to which only the participant and the principal investigator had access. It was used to describe in detail issues about their working life in the ACCs, with weekly monitoring through meetings via chat.

### Analysis

STATA17® software was used for descriptive statistics. In the qualitative approach, the NVivo® software was used in two stages, the first from the thematic analysis based on units of analysis where codes and categories were assigned with the constant comparison method until data was saturated. Then, axially they are grouped by themes and patterns that relate those units of analysis, reviewed, diagrammed and discussed in depth by the researchers to reach a consensus [25] and the second from a structural analysis based on the Labov model and Waletzky who develops 7 elements of a story stimulus, summary, orientation, complicated actions, evaluation, resolution and code [26]. The triangulation of the data was of an interactive nature between expert pairs using diagramming and discussion, and it was also returned to the key informants for verification.

After completing the analysis of quantitative and qualitative data, the data were integrated in two moments [27, 28]: Moment 1. Consolidation of quantitative and qualitative data. In this process, the convergences or links, the divergences and the complementarity were sought on the evidence that the evaluated elements of the QWL show and the relationship with the organizational culture of the HWF in the ACCs.

Moment 2. Analysis in which themes/categories are named with a diagrammed combined matrix or joint displays [29], taking into account the general theory of stress, that of social capital, the theory of motivation, the model of fatigue or satisfaction out of compassion, and the theory of cultural or adaptationist ecology, based on an integrative model. That is, the integration of the data capitalizes on and enhances a better understanding by combining data and sets of findings [30] to reveal inferences and information on the QWL of the HWF from the two methodological approaches.

The comparison between qualitative and quantitative results is presented as proposed by Morgan [31]. The convergent results indicate that the responses are valid for each method in the case of the QWL measurement, complementary when the questions were answered by each additional method (CopSoQ, ProQoL and turnover intention) or divergent when the responses were contradictory between methods. and instruments other than QWL. For this last category of findings, the intention is to open a dialogue that generates alternative research opportunities to address these differences [32]. For the development of the study, scientific rigor was followed in mixed methods research: interpretative rigor, design quality and legitimacy [33].

## Results

### Quantitative approach

Of 209 participants, the percentage for women was 71.8%, see Table 1, and the mean age was 33 years (minimum 20; maximum 65 years); the majority of staff that work in the ACC is nursing at 53.2%.

**Table 1:** Distribution of professionals who work in the ICU of Valle del Cauca - Colombia during 2020–2021 (*n* = 209)

	Gender					
Profession	Male	%	Female	%	Total	Total %
Nursing assistant	14	6.7	34	**16.3**	48	**23.0**
Nurse	25	12.0	80	**38.3**	105	**50.2**
Medical specialist	7	**3.3**	2	1.0	9	4.3
General practitioner	10	**4.8**	8	3.8	18	8.6
Physical therapist	2	1.0	13	6.2	15	7.2
Respiratory therapist	1	0.5	13	6.2	14	6.7
Grand Total	59	28.2	150	**71.8**	209	100

*Source* Own work.

For the total number of participants, the average number of years of study, including primary school, was 17.6 years (± 5.12); the average time in years of working seniority was 4.8 (± 5.6), and the average hours worked per week was 50.6 (± 10.3). Other population characterization data are found in Table 2.

**Table 2:** Characteristics of the health workforce of the CCAs of Valle del Cauca - Colombia 2020–2021 (*n* = 209)

Characteristic	Modality	Frequency	Percentage
Civil status	Married	36	17,24%
	Divorcies	7	3,34%
	Single	**130**	62,20%
	Free Union	35	16,74%
	Widower	1	0,47%
Level of study	Specialization	27	12,91%
	Master’s degree	9	4,30%
	PhD	1	0,47%
	Undergraduate	**124**	59,33%
	Technical	48	22,95%
Position in the company	Assistance	**182**	87,08%
	Assistance and administrative	21	10,04%
	Administrative	6	2,87%
Schedule	Rotating shift (morning- afternoon or night)	**187**	89,47%
	Office hours (8 h a day)	18	8,61%
	Rotating shift except nights	2	0,96%
	Fixed shift at mornings	1	0,48%
	Fixed shift at night	1	0,48%
Causes of disability in the last year *	COVID 19	35	76%
	Others**	11	24%

*Source* Own work.

*22% Twenty-two% reported illness in the last year.

** Cervical injury caused by mobilization of patients, lumbago, vertigo, kidney stones, ectopic pregnancy, fibromyalgia, and eye and urinary infection.

On the results the HWF is unsatisfied in four out of four dimensions of the QWL-GOHISALO with an average of 9.8 (± 1.7) for Integration into the workplace (59.8%), 9.6 (± 2.1) for Personal development (64%), 19 (± 3.2) for Satisfaction at work (74.2%) and 5.5 (± 2.1) for Free time management (76.6%). To see the comparative percentage distributions between these dimensions, Table 3.

**Table 3:** Distribution of the QWL-GOHISALO scores of the WHF in the ACC of Valle del Cauca– Colombia during 2020–2021 (*n* = 209)

Dimension	Satisfaction (%)	Insatisfaction (%)
Institutional Support for work	**68**	32
Safety at Work	**66.5**	33.4
Integration into the workplace	40.2	**59.8**
Satisfaction at work	25.9	**74.2**
Well-being achieved through work	**68**	32
Personal development	36	**64**
Free time management	23.4	**76.6**

Own elaboration.

Regarding psychosocial risks, of the 15 dimensions measured, the most unfavorable for health are the pace of work (84%), insecurity in working conditions (68%), quantitative requirements (63%), job insecurity (58%), role conflict (45%) and justice (40%). The dimensions with the most favorability were the possibility of development (94%), sense of work (85%), clarity of role (67%), double presence (55%), emotional demands (40%), quality of leadership (35%) and vertical confidence (34%). Regarding the dimensions that were classified as intermediate, there are influence (39%) and predictability (37%).

Regarding satisfaction due to compassion– SC, more than half of the ACC health workforce is classified as high, which is equivalent to 67%, with an average score of 43.5 (SD = 5.3). Both burnout (BO) with an average score of 20.8 (SD = 7.7) and the secondary trauma scale (STS) with an average score of 17 (SD = 9) are classified on average between 46.4% and 26%, respectively. Two professionals are classified as “high” for BO and STS.

Most of the HWFs reported a low intention to rotate to another service (67%), to another institution (56%), to migrate to another city (63%) or to change to independent work (56%). In contrast, 66% reported a high intention to migrate to another country.

### Qualitative approach

With narrative research based on hermeneutics through details expressed by the participants’ own words, a route was woven between the structured stories to men and women from different professions (3 doctors, 4 physiotherapists, 2 nurses and 1 nursing assistant) who work in both public and private ACCs to build knowledge.

In this part of the manuscript, two main themes are presented that emerge in the data analysis and that are not evaluated through the instruments applied in the quantitative approach but that emerged from the qualitative approach. The first “Discrimination and harassment as normalized practices” and the second “The faceless spirituality of THS in the UCIA.”

An additional theme emerges “multidimensionality of the QWL” expressed by the 7 dimensions (categories) that make up the instrument of the quantitative approach institutional support, job security, integration into the job, job satisfaction, well-being achieved through work, employee personal development and leisure time management, which are inserted in the analytical matrices of the approach to the integration of methods. See supplementary material at Mendeley Data [34].

#### Discrimination and harassment as standard practices

Some reports from the participants show tolerance of various types of discrimination among them based on race, gender, age and profession, the latter perpetuated through practices of workplace harassment. An example of this situation is recounted by Amatista regarding her ethnic-racial group, who mentioned a broken voice and tears in his eyes:

“…a relative of a patient told me: I do not like being treated by people of your race, your color, I am ah! (Silence, she stutters) The truth is, I did not know whether to cry, laugh, shout, hit him… that shift I cried all 7 hours! I have never felt so humiliated, trampled on, beaten… because of my color! (M2(40)E).

Those who witnessed the situation chose to ignore what was happening, and it is evident how these become repeated behaviors framed in patrilineality. Said tolerance to something that is known to be wrong becomes a type of adaptation of the human being to the environment based on a patriarchal system, without sisterhood and that, in a few words, allows discrimination.

Impunity and “adapting” to living in a state of discrimination is a reality for many women, as Jade relates:

“It always happens to me, because I say that it is like the combination of the 2 things, being a woman and being black (pleasant laughter) it is very funny because they arrive and it is… and the doctor?! So sometimes… when I do not care about anything I let them hang around and when the boss says there’s the doctor sitting, then I, oh, is it that women doctors do not exist? and black worse!! I tell them… but then if I do not have a gown on I’m not a doctor, if I do not have something that says doctor… I’m not, but it is something I finally learned to be with"… “some say I’m very girl, that I am a woman” (M9(30)MD).

The foregoing highlights a fragmented being: ecological “me versus nature versus technology”, a social rupture “me versus the other” and a spiritual crisis “me versus me” that requires a frontal approach that involves ALL parties in search of compassion from and for the HWF.

#### Faceless spirituality

Hospices evolved from the public to the private within the framework of a capitalist society, which made it evident that the HWF, when seeing their physical and/or mental health threatened, frequently adapts and uses coping strategies; one of these is spiritual, and the other is religiosity, which helps reduce psychological distress, balance, and promote healing states. In the case of the health workforce, these strategies can promote quality of life at work, although they are not always recognized, well managed, or externalized.

A participating professional pointed out: “…I believe in angels but I’m not much of talking about that spiritual part with family members because it is something that we have always had to respect their beliefs, the taboo and the fear of offending them or being reckless. I do not want people to perceive what is not…” (M9(30)MD).

Those who work in the ACCs know that religion and spirituality have been absent from the discussions on the QWL of the HWF in the last 15 years. The interaction of the spiritual with the scientific has undergone modifications around the technological evolution from the rational to believe only in what can be proven, which is not compatible with spirituality, since it cannot be seen, proven or validated directly, so it is simply marginalized or downplayed.

Contradictorily, at present in this conflict, quality parameters are established in care from the holistic perspective, but without fully incorporating care in the spiritual dimension, both for patients and for the people who make up the health workforce.

The HWF is integrated into a capitalist society that encourages them to be productive by focusing on material aspects and not on holistic professional development in the CAC. The impact of technology has made the open and shared management of spirituality difficult and results in processes where health has become a commodified good.

### Mixed methods integration

This consolidation process in terms of convergence, complementarity and/or divergence resulted in the findings combined with two main topics, the first, “What the HWF that works in the ACCs gains”, see Table 4 and the second, “The sacrifices of the HWF in the exercise of their profession” see Table 5.

**Table 4:** Results of the convergent parallel design “what the HWF that works in the ACCs earns” to interpret the QWL of the HWF in a region of Colombia 2020–2021

Quantitative results	Qualitative results	Result of combined findings
Most of the HWF in ACC are satisfied with institutional support, job security and well-being achieved through work	• Training and permanent updating • Experience time • Balance between salary and work effort • Endowment and inputs • Disconnection with work • Individual/group celebrations and rituals • Institutional support • Technology evolution • Plant contracting • Feeding during shifts	Convergent
The intention of the HWF to rotate to another service, another HSPI, another city or to independent work is low	• Thoughts of leaving the service • Situations that favor the intention of breaking	Divergent
Most of the HWF have a favorable exposure to psychosocial risk for health in the dimensions of leadership quality, possibility of development, sense of work, clarity of role, double presence, vertical trust and emotional demands	• Personal spirituality present but not visible • altruistic • Committed • Acceptance of hierarchies • Personal satisfaction from the duty fulfilled • Highly trained and efficient • Disciplined • Compliance with protocols and institutional regulations • Permanent training opportunity with technological updating	Complementary
The HWF of the ACCs has a high satisfaction for compassion	• HWF compassionate with patients, but not always with the colleagues • Satisfaction of saving lives •Empathy with patients and work team	Complementary
The HWF of the ACCs has low compassion fatigue (Burnout - BO and secondary trauma stress - STS)	• Dehumanization of care • ICU with open or closed doors • Suffering and loss of patients managed through follow-up with therapists • Mechanisms of psychological protection against pain or periods of crisis • Work under pressure is best done in a team	Complementary

*Source* Own work.

Regarding the factors that positively influence their QWL, some hypotheses are proposed as to why they continue working at the ACC despite having other dimensions in which they are not satisfied, which will be pointed out in the following section. The quantitative results regarding the dimensions of the QWL that have approximately 58% of the HWFs satisfied are related to the link that exists with the health institution where they work, described by those elements such as the acceptance of supervision by their superiors, the opportunities of promotion, autonomy and work processes themselves in the current position they hold.

This description converges with the stories of Zafiro and Amatista regarding technology training, length of experience, balance between salary and work effort, resources and inputs, and disconnection from work:

“This Saturday I spent the night, it was a little complicated because patients came to me to intubate, I rested little, fortunately I have a good team to work with and we squared up the room quickly” (M4(33)FT).

“…it is my first day as an ICU coordinator, bringing all these experiences and knowledge that I have obtained over 9 years in critical care… I feel simply happy! Now comes a week of meetings and little by little strengthening my new role, I just hope it takes me on the best path, and prove to myself that it was the best decision” (M1(40)E).

“For me, hierarchies are leadership, they must exist, they are the people who have clear objectives and who guide people to achieve those objectives” (M9(30)MD).

These factors are complementary to the 60% favorability in the health of the ACC workforce regarding the risk of psychosocial exposure within the framework of their ability to understand the situation of others (patients, relatives or coworkers who suffer) and generate compassion for them, as well as in the stories that show the double shifts to earn more money, established by the shortage of personnel in times of COVID-19 or without it, putting the relationship with their domestic responsibilities to the limit. Aquamarine and Sapphire refer:

“Today I extubated a patient! It made me very happy because it had been more than two months since I had extubated a patient. Almost all the patients have died… Almost all of them obese, with comorbidities of hypertension/diabetes and they have become complicated! That has kept me happy this turn, I have no desire to leave (Aquamarine smiles). It is very emotional to know that lives can begin to be saved even with the shortage of medicines and supplies due to the pandemic” (M10(28)FT).

“For me, the most positive thing is always when I manage to get a patient through, that for me is like an adrenaline rush” (M9 (3)MD).

Opportunities to develop skills and knowledge become stimulating factors that give meaning to work through their occupational profiles, according to the functions they perform in their positions. the stories of Caicedonia and Esmeralda that support a complementary finding when integrating the results:

“During the pandemic we have had to learn many things and the institution has been in charge of training us” (H8(27)MD).

“When they train you feel empowered by what you are doing” (M10(28)FT).

“Not everyone has the same thirst for knowledge” (M4(33)FT).

However, when reviewing other reports, divergence is found, although health institutions develop training activities, considering that the work environment in the ACCs requires the use of technology and that it advances by leaps and bounds, not all of them consider it a priority for their health workforce and they are not enough or needed.

This could be because the institutions do not include a training program that considers the academic level, motivations, age, and seniority of the HWF to develop the activities that, in addition, takes into account the different learning systems immersed in the andragogy of care with the respective monitoring of the learning process. This statement is supported in the stories of Opal and Amethyst:

“It is a quite big challenge because many times the technology should come accompanied by training and the training is not always the best considering that we do not have much time available” or “I have colleagues who still struggle with the computer to open the program to be able to making nursing notes, for example, does not help them, so they become stressed, despite the training” (H3(36)E).

“I have a staff of 30, 40, 50 and we are not all strong in handling an ultrasound machine or other processes that require the use of technology. We do not have the same learning ability, so if you as a company or institution do not provide education to handle technology, the technology is going to stay there and not everyone is going to take advantage of it. The technologies themselves are good but the institutions do not invest in the education of personnel” (M2(40)E).

Additionally, it is likely that the high compassion satisfaction represented by 67% of the HWF who participated in this study with low compassion fatigue by 63% is reinforced by the factors described above, giving the most positive possible result described in the literature (16) for the HWF that is satisfied in three of the seven dimensions of the QWL, probably because they receive reinforcement from their work in a positive way. This HWF has no significant concerns about being "stuck" or inexperienced to do their job, either as individuals or within their organization, becoming influential to their peers and their institution. HWF may be welcomed by patients who attend it and by those who seek its help.

Divergence is found between the low intention to rotate or leave the profession because in the reports the HWF refers that they do have thoughts of changing their workplace to another health institution where they have the possibility of improving their quality of life. Like for example when Sapphire says:

“A colleague went on vacation, and they loaded the rest of us with work, it’s really very heavy right now. I have even thought about quitting… it really causes me a lot of stress” (M4(33)FT).

The results are diverse, with some consistent with the qualitative and quantitative findings and others divergent. It stands out that the health workforce is highly committed, resilient, and adapts by providing an organizational culture that resists leaving the ranks of the ACCs for a limited time.

This section shows significant aspects for the HWF that works in the ACCs of the present study due to being dissatisfied with the QWL evidenced in 69% of the labor force with respect to 4 of 7 of its dimensions, which in turn are related to 71% of the HWF who have exposure to unfavorable psychosocial risk for health. The data are convergent when the imbalance is evident between the level of preparation of the HWF and the respect of the contractual rights of connection to the HSPI with a compensation system that shows concern for future work regarding salary type, forms of payment, hours, and job position, with consequences on their job stability, as reflected in the combined analysis.

**Table 5:** Results of the convergent parallel design “the sacrifices of the HWF in the exercise of their profession in ACC” to interpret the QWL of the HWF in a region of Colombia 2020–2021

Resultados cuantitativos	Qualitative results	Result of combined findings
Most of the HWF in ACC are dissatisfied with the integration into the job, job satisfaction, personal development of the worker and free time management	• Discrimination by profession • Adverse events • Delays in payment of wages • Ignorance of labor policies • Gender discrimination • Double work • Shortage of advanced technology • Payment of salary glosses • Salary differences • Retaliation	Convergent
The intention of the HWF to migrate to another country is high	• Clear signs of wear with verbalization of boredom or dissatisfaction with working conditions • Verbalization of the desire to migrate to another country	Complementary
The majority of the HWF obtained an unfavorable psychosocial risk exposure score for health in the rhythm of work, insecurity in working conditions, quantitative demands, job insecurity, role conflict, justice, influence and predictability	• Shortage of FLS or minimal hiring of personnel to reduce organizational costs • Overload of those who are linked • Uncertainty and labor instability due to types of contracting outsourced or on terms • Broken employment relationship between the organization and the FLS due to outsourcing • Conflict management is not always fair • FLS adapted to working conditions due to the need to receive a salary • The organizational culture allows certain degrees of autonomy, but with role conflicts • Invisible spirituality toward patients and their families	Complementary

*Source* Own work.

It was also found that there is a dedication to work almost exclusively juxtaposed to a majority of the HWFs with a global feeling of displeasure with aspects associated with the use of their skills for activities that sometimes do not belong to their current position. As Esmeralda expresses it:

“We must take care of the oxygen cylinders, that doesn’t seem to be my function because in the other institutions where I have worked that is done by the messenger, stretcher bearer… in charge of medicinal gases… I have to go down several floors and walk several floors to the central pharmacy carrying that oxygen cylinder, see if they can change it there for a new one and carry it up again to leave it full for my partner. So be tenacious, because sometimes there is no… you miss the trip, you return with nothing! But thank God, today there were” (M10(28)FT).

The work activity itself does not motivate the staff in relation to the scope of their achievements and is related to unfavorable expectations of job growth and intangible benefits under situations that challenge their own physical, mental, and spiritual health daily with a frequent sensation of being at risk. In this regard, Rubí relates:

“Right now I am in treatment because I had an anxiety crisis… it started in July and it was very strong but I did not want to continue the medical and pharmacological treatment with the psychiatrist and about 15 days ago the anxiety crisis recurred” (M5(25)FT).

And Sapphire expresses:

“…communication is very difficult, especially with doctors, the more professionals the more difficult… there is a very ugly gap!!…if it is difficult between the professional and the specialist, now imagine yourself with an assistant” (M4(33)FT).

Regarding the administration of free time, the HWF exercises control over what he does with himself; however, the way in which the institution schedules the shift rotation of health personnel influences the availability of time, which causes a fracture between the work situation in relation to the enjoyment of life with family and friends. This situation may explain the frequent social and family isolation that those who work in the ACCs are subject to. As Lapis Lazuli and Amethyst relate:

“Sometimes you don’t notice it, but you take your work tasks home"… “it affects your family, the social role that you also fulfill at the community level… my wife tells me that I arrive every days when during the week I am an ogre and on the weekend I am loved” (H1(40)MD).

“They affect you in the sense that you don’t have time and that you live stressed, you always live tired and sleepy, then it gets to the point where you live at work and go home to sleep” (M2(40)E).

Other results related to spirituality or aspects of discrimination or workplace bullying, described in the qualitative section, when analyzed, are divergent from the data resulting from the measurements with the use of QWL instruments, exposure to psychosocial risk and satisfaction/compassion fatigue, because these instruments do not incorporate items that demonstrate these situations in the work context that undoubtedly influence the QWL of the HWF in the ACCs. The ProQoL instrument has an item related to spirituality, but when making a global score aimed at fatigue or satisfaction due to compassion, it loses relevance in this regard.

## Discussion

The mixed-methods integration findings of this study are discussed considering the emerging scientific literature on the use of this methodology in relation to the HWF of the QWL. The little evidence found with a mixed design, which although it uses methods that complement each other, does not integrate the methods for its interpretation [10, 36].

This discussion focuses on exposing the principles and relationships that the findings indicate with practical significance and on the implications for practice based on a theoretical input from an adapted model of QWL that emerged from the results; it also shows the limitations of this study.

It is likely that the high compassion satisfaction represented by 67% of the HWF who participated in this study with low compassion fatigue by 63% is reinforced by the factors described above, giving the most positive possible result described in the literature [16] for the HWF that is satisfied in three of the seven dimensions of the QWL, probably because they receive reinforcement from their work in a positive way. This HWF has no significant concerns about being “stuck” or inexperienced to do their job, either as individuals or within their organization, becoming influential to their peers and their institution. HWF may be welcomed by patients who attend it and by those who seek its help.

The **integration** between the qualitative and quantitative approaches in this study results in the convergence that represents its credibility, while the **satisfied** dimensions of the QWL lead to explaining that a motivated, resilient, and committed HWF to its work, even during the crisis caused by the pandemic, shows that you have more to gain than lose by remaining in service and providing quality care. In contrast, some negative factors are related to dissatisfaction with integration into the job, personal development and free time management that converge with the qualitative findings reported by the participants.

Taking into account that these reports complement the unfavorable finding for health when situations arise that do not allow the HWF the opportunity to adapt to changes in work associated with shifts that represent the availability of time for activities other than work depending on the states of alert generated not only by the pandemic but also by emergency and/or disaster events that may occur in the local or regional context, due to anthropogenic causes caused by human activity such as festivities called “fairs”, pollution, strikes violent or natural, for example, floods due to rain or forest fires, or when the IPS links a recently graduated HWF with no experience or specialization in critical care.

In some cases, it is necessary to leave their city of origin and family to join the labor market without allowing them preparation time with an adequate transition between starting work called the novitiate and gaining expertise [37].

With what was presented, analyzed and interpreted, it is observed that there is an imbalance between family - social life - work - free time associated with situations of workplace violence based on gender and race, as found in this investigation, which is consistent with the results of similar studies [35, 38–42].

It is found that the narratives explain in a deep and specific way the aspects that have been valued on the QWL through quantitative instruments. This can facilitate differentiated planning of potential improvement interventions and strengthening of what is working well.

The tracking of qualitative antecedents on QWF of the HWF showed that those found converge with the quantitative reports, which indicates the need to carry out more research with integration of methods to provide elements to establish improvement strategies from the institutions and from the individual [43].

The positive aspects for the quality of life at work have a limit and even more when the HWF of the ACCs shows that there are other negative dimensions that weigh and call into question their permanence in this care area because it is reflected in their intention to rotate, to leave their profession or to migrate to another country in search of a way out toward a better quality of life, which shows a divergence, according to the results of the quantitative approach, given that the information collected in the interviews and newspapers shows the contrary, with signs of recurrent thoughts about leaving the service to go to another institution or finally to migrate to another country, which coincides with other studies [44–46].

The complementary findings arise from the favorability or not for health from the prevention of psychosocial risks, the presence of compassion satisfaction and the low presence of compassion fatigue that predict QWL and that are explained from the narrative that is immersed in this construct in search of health promotion in work environments.

These complementary results are expected since there is empirical evidence that has been accumulating separately by topic of interest, relating QWL with psychosocial risk, personality traits, workplace violence, compassion fatigue, and gender, among others [47–52].

### Implications for ACC practice

The knowledge from the integration of methods provides a broader and clearer picture both for future research and for the improvement of QWL programs.

**Fig. 1:**
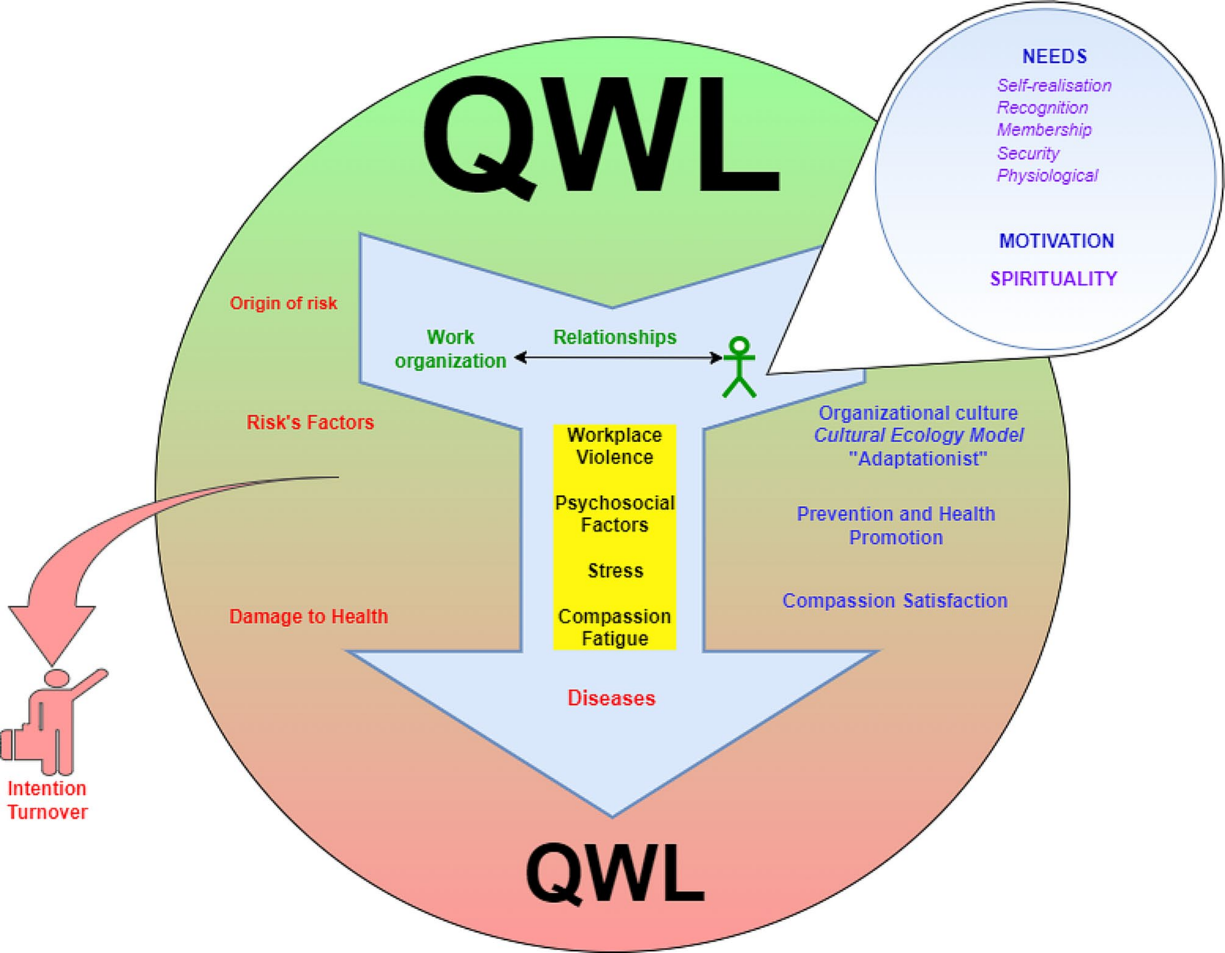
Comprehensive model of the QWL of the HWF in the ACCs. Own elaboration

The following model has been adapted and supplemented, taking into account this integration of mixed methods. See Fig. 1 adapted from Moncada et al. [53], explains how the QWL that is satisfied, green background, is present in individuals as part of the expectation when entering as a worker in a health institution; However, this satisfaction can be improved/maintained or not until reaching dissatisfaction, represented in the change of color towards the red background as a transition in which negative factors, yellow background, such as violence in the workplace can occur, psychosocial factors harmful to health, stress and compassion fatigue that includes burnout and secondary trauma (these can occur at the same time or individually with different levels of impact on workers).

Once the individual begins work, it must be taken into account that he or she has personal needs that include physical, psychological, social and emotional aspects that are energized through his or her personal motivations; As well as, over time each worker builds relationships within the work organization that may or may not influence the quality of work life and the responses to possible difficulties that may arise in the future.

The arrow with two tails represents the two possible paths, the left one with red letters that shows the origin of the risks, the factors that trigger them and that, if not intervened by the organization, the worker may well rotate and even leave the profession for personal decision as shown by the arrow and the individuals who enter or leave the organization, but diseases that pose a risk of death can also be triggered. The right side, blue letters, proposes some ways in which these risks are compensated through adaptive processes that are embedded in organizational culture, health prevention and promotion, and compassion satisfaction.

Situations that can be greatly reduced with QWL activities and programs based on health promotion and that generate satisfaction out of compassion or improve existing ones.

### Study limitations

The present investigation has some limitations. First, a cross-sectional design was used, and a longitudinal study would have been necessary to prove that the variables analyzed have a cause‒effect relationship. Second, social desirability bias may be present when answering the instruments. Third, other variables have not been analyzed, such as training in coping skills to handle situations of workplace violence, and in the available databases of the scientific literature consulted, no articles were found that address QWL in an integrated manner with the use of mixed methods. Fourth, there was no direct face-to-face contact with the participants due to the isolation imposed by the pandemic. The management and execution of the field work was carried out through Information and Communication Technology - ICT.

## Conclusions

The investigation with integration of mixed methods allows us to give clarity and specificity on the aspects that must and have to be contained in the improvement plans of the QWL of the HWF, showing the **complementarity** of the approaches and the use of various instruments that resulted in negative tendencies regarding integration into the workplace, job dissatisfaction and an important **convergence** regarding the inadequate administration of free time. In addition, situations of discrimination, adverse events, delays in salary payment and ignorance of labor hiring policies are made **visible**.

The positive trend that **converges** with the two approaches is that of safety at work and well-being achieved through work, embodied in the constant updating of technology and care protocols, experience time, balance between salary and work effort, staffing and supplies, and disconnection with work.

On the other hand, the findings related to the intention to rotate or migrate to another city/country resulted in **divergence**, since the quantitative results report that it is low; the qualitative approach shows another reality, which partly explains the lack of retention and motivation of the HWF in the ACCs of the region.

Last, there is evidence that intervening in the factors associated with the QWL of the HWF would initially benefit the staff, the HSPI and indirectly the health system in general. Some of these benefits that will be reflected in the health workforce would be positioned to promote its development, resulting in more motivated staff to better perform their duties, reduce the possibility of absenteeism, intention to rotate, express complaints from both workers and users and increase both QWL satisfaction and efficiency and productivity.

It was found that there is no organizational culture aimed at periodically measuring the QWL of the HWF. Knowing the labor reality will make it possible to have indicators that, when taken into account, facilitate and make sustainable the implementation of comprehensive programs focused on this issue.

### Electronic supplementary material

Below is the link to the electronic supplementary material.

Supplementary Material 1

Supplementary Material 2

## Data Availability

The data sets generated and/or analyzed during the current study are not publicly available because the ethics committee that approved the fieldwork required a clause in which each participant is asked whether or not they accepted that the obtained and coded data be published and not all agreed, but they are available from the corresponding author upon reasonable request.

## Abbreviations

**QWL**: Quality work of life
**HWF**: Health work force
**HSPI**: Health Service Provider Institutions
**ACC**: Adult critical care
**ProQoL**: Professional Quality of Life Scale
**CopSoQ**: Copenhagen Psychosocial Questionnaire
