# Supplementary material for: Quality of work life for health professions in Colombia’s adult critical care: An integrative analysis
Source: BMC Health Serv Res. 2024 May 3;24:582. doi: 10.1186/s12913-024-10780-z (PMC11071144; doi:10.1186/s12913-024-10780-z)
Supplement: Supplementary file 1 — Supplementary Material 1 [file 12913_2024_10780_MOESM1_ESM.pdf]

GUIDING QUESTIONS FOR SEMI-STRUCTURED INTERVIEW ON QUALITY OF WORK LIFE

Taken and adapted from Vidal-Blanco et al. (1)

A. Presentation/personal identification of the interviewee

Age, years of experience at Work, years in the current Intensive Care Unit.

B. Context of the work environment

a. What is your opinion about the way decisions are made within the intensive care unit? What are the interactions of the work team in your unit? How are communications between Human Talent in Health?
b. How does the use of technology influence your daily activities and the care of your patients?
c. What is your opinion about your current working conditions?
d. How do these working conditions affect you, your family, and your patients?
e. How do regulatory policies in the Work of Human Talent in Health in your country influence your professional actions?
f. What are the activities that protect you or that you use to feel more comfortable with your work?

C. About Quality of Work Life

g. What situations affect the Quality of Work Life on a normal day in the Intensive Care Unit where you currently work? How do you feel?
h. For you, what aspect predominantly influences Quality of Work Life at and for you, what is quality of life at Work?
i. What do you consider to be the best ways to promote Quality of Work Life in the Intensive Care Unit where you currently work?

REFERENCIA

1. Vidal-Blanco G, Oliver A, Galiana L, Sansó N. Quality of work life and self-care in nursing staff with high emotional demand. Enferm Clin [Internet]. 2019 May 1;29(3):186–94. Available from: https://doi.org/10.1016/j.enfcli.2018.06.004

PREGUNTAS ORIENTADORAS PARA ENTREVISTA SEMIESTRUCTURADA SOBRE CALIDAD DE VIDA EN EL TRABAJO

Tomada y adaptada de Vidal-Blanco et al.(1)

A. ***Presentación/identificación personal del entrevistado***

Edad, años de experiencia en el Trabajo, años en la Unidad de Cuidado Intensivo actual.

B. Contexto del entorno en el Trabajo

a. ¿Qué opinión tienes acerca de la forma en que se toman las decisiones dentro de la unidad de cuidado intensivo? ¿Cómo son las interacciones del equipo de trabajo en tu unidad? ¿Cómo son las comunicaciones entre el Talento Humano en Salud?
b. ¿Cómo influye el uso de la tecnología en tus actividades diarias y en la atención a tus pacientes?
c. ¿Qué opinión tienes acerca de tus condiciones laborales actuales?
d. ¿Cómo te afecta a ti, tu familia y a tus pacientes estas condiciones laborales?
e. ¿Cómo influyen las políticas de regulación de tu país en el Trabajo del Talento Humano en Salud y en tu actuar profesional?
f. ¿Cuáles son las actividades que te protegen o que utilizas para sentirte más cómodo/a con tu trabajo?

C. ***Sobre la Calidad de Vida en el Trabajo***

g. ¿Qué situaciones afectan la Calidad de Vida en el Trabajo en un día normal en la Unidad de Cuidado Intensivo donde trabajas actualmente? ¿Cómo te sientes?
h. Para ti, ¿Qué aspecto influye predominantemente en la Calidad de Vida en el Trabajo y para ti qué es calidad de vida en el Trabajo?
i. ¿Cuáles consideras son las mejores formas de promover la Calidad de Vida en el Trabajo en la Unidad de Cuidado Intensivo donde laboras actualmente?
